# Crossover Effects of Estrogen Receptor Status on Breast Cancer-Specific Hazard Rates by Age and Race

**DOI:** 10.1371/journal.pone.0110281

**Published:** 2014-10-21

**Authors:** Yu Ren, Dalliah M. Black, Elizabeth A. Mittendorf, Peijun Liu, Xu Li, Xianglin L. Du, Jianjun He, Jin Yang, Kelly K. Hunt, Min Yi

**Affiliations:** 1 Department of Surgical Oncology, The First Affiliated Hospital of Xian Jiaotong University, School of Medicine, Xian, China; 2 Department of Surgical Oncology, The University of Texas MD Anderson Cancer Center, Houston, Texas, United States of America; 3 Department of Translational Medicine, The First Affiliated Hospital of Xian Jiaotong University, School of Medicine, Xian, China; 4 Division of Epidemiology Human Genetics, and Environmental Sciences, The University of Texas School of Public Health, Houston, Texas, United States of America; 5 Department of Medical Oncology, The First Affiliated Hospital of Xian Jiaotong University, School of Medicine, Xian, China; University of North Carolina School of Medicine, United States of America

## Abstract

**Background:**

Previous studies found that the risk of breast cancer–related death is greater in estrogen receptor (ER)-negative disease than in ER-positive disease within 5 years of diagnosis, but greater for ER-positive disease than for ER-negative disease more than 5 years after diagnosis. This phenomenon is referred to as ER-positive and -negative crossover. Our aim was to evaluate this crossover by determining the timing of the hazard of breast cancer death by patient, clinical, and tumor factors.

**Methods:**

Patients with breast cancer diagnosed between 1990 and 2005 were identified from the Surveillance, Epidemiology, and End Results database. The cohort was evaluated by age at diagnosis, race, tumor ER status, tumor and nodal stage, and tumor grade. Disease-specific (DS) hazard rates were calculated.

**Results:**

Of the 439,444 patients identified, 77.5% had ER-positive disease. Overall, ER-negative to ER-positive DS hazard rates crossed between the years 7 and 8 after diagnosis. Earlier crossover was linked to black or Hispanic race, young age (<40 years), or tumors that were larger, higher grade, or affected the nodes. Young black (<40 years) patients who had a T3/T4 tumor with positive nodes, grade III or undifferentiated, had the earliest crossover, in year 4.

**Conclusions:**

The timing of crossover of death hazard for ER-positive and ER-negative disease varies by clinical and tumor factors. These findings may help guide recommendations regarding the duration of endocrine therapy for patients with ER-positive cancer.

## Introduction

Worldwide, two-thirds of breast cancer cases present as estrogen receptor (ER)-positive disease [1], [2]. With appropriate local therapy and targeted systemic endocrine therapy, early recurrence and survival outcomes for ER-positive breast cancers are excellent. Five years of adjuvant endocrine therapy is the standard of care for patients with ER-positive, early-stage breast cancer [3]. Despite excellent early outcomes in ER-positive patients treated with endocrine therapy, some patients with ER-positive disease experience recurrence and die from breast cancer more than 5 years after diagnosis [4]–[7]. In fact, previous studies found that while the risk of recurrence or death due to breast cancer is greater for women with ER-negative breast cancer than for those with ER-positive breast cancer within the first 5 years after diagnosis, the risk is greater for ER-positive than for ER-negative breast cancer after the first 5 years [7]–[9]. This phenomenon is referred to as ER-positive and -negative crossover.

In part because of this crossover effect, there is significant interest in defining the appropriate duration of endocrine therapy in patients with ER-positive breast cancer. In a meta-analysis, the Early Breast Cancer Trialists' Collaborative Group (EBCTCG) evaluated 20 trials that included 21,457 patients who were randomized to receive tamoxifen or not. This combined analysis found that 5 years of tamoxifen reduces the 15-year risk of breast cancer relapse and death [10]. The question of whether tamoxifen taken for a longer duration would be of benefit was addressed in the Adjuvant Tamoxifen: Longer Against Shorter (ATLAS) trial. That study, which included 6846 patients, found that continuing tamoxifen to 10 years reduces the risk of recurrence and death compared to stopping at 5 years in patients with ER-positive cancer [11].

The aim of this study was to evaluate the timing of the crossover effect by different patient, clinical, and tumor factors, including age at diagnosis, race, tumor and nodal stage, and tumor grade.

## Patients and Methods

The Surveillance Epidemiology and End Results (SEER) database of the National Cancer Institute was used to identify patients with a primary tumor site coded as C50.0 to C50.9 (breast) between 1990 and 2005. Data were obtained from all 18 U.S. cancer registries participating in the SEER program using SEER*Stat software version 8.0.2 under a data user agreement (http://seer.cancer.gov/seerstat). Patient records/information was anonymized and de-identified prior to analysis. The First Affiliated Hospital of Xian Jiaotong University ethics committee review board approved this retrospective study. ER status was categorized as positive (>1%) or negative, and patients with unknown ER status were excluded.

### Statistical Analysis

Disease-specific (DS) hazard rate was calculated from the date of diagnosis to the date of breast cancer-related death, date last known to be alive, or November 30, 2010, which was the last follow-up date for SEER data. Hazard rates of DS curves were calculated using the graph smoothed hazard estimate. Patients who were lost to follow-up or who survived beyond November 30, 2010, were censored.

The univariate association of each potential prognostic factor with DS hazard rates was calculated. Multivariate Cox proportional hazards models without violation of the proportional hazards assumption were used to determine the influence of patient and tumor factors of known or potential prognostic value on DS hazard rates with backward stepwise exclusion of factors.

The final regression model was chosen based on the clinical and statistical significance of the predictors. The 5- and 10-year predicted probability of death by breast cancer was calculated for each patient using the Cox regression model underlying the nomogram. Model performance was quantified using Harrell's concordance index.[12] The discriminative ability of the model was assessed using the concordance index (C-index) for comparative purposes with the literature as well as with the concordance probability estimate because of the high degree of censoring in the data.[13] The concordance probability estimate (CPE) can range from perfect concordance (1.0) to perfect discordance (0.0). Stata SE version 12.0 statistical software (StataCorp LP, College Station, TX) and R 3.0.2 (http://www.r-project.org/) were used for statistical analyses. All tests were two-tailed, and statistical significance was set at P<0.05.

## Results

Of the 439,444 breast cancer patients identified in the SEER database, 77.5% had ER-positive disease. Baseline patient, clinical, and tumor characteristics are shown in Table 1. Median follow-up time was 7.6 years; more than 48% of patients had follow-up monitoring lasting longer than 5 years, and 29% had follow-up monitoring longer than 10 years.

**Table 1:** Baseline patient demographic and clinicopathologic characteristics.

Characteristic	No. of patients (%)
Race	
White	339307 (77.8)
Black	37785 (8.7)
Hispanic	31102(7.1)
Asian/Pacific Islander	27990 (6.4)
Age at diagnosis, years	
Mean (Median)	61 (61)
<40 years	25417 (5.8)
≥40 years	414027 (94.2)
Sex	
Female	438972 (99.4)
Male	2759 (0.6)
T-stage	
Tis	209 (0.1)
T1	253849 (63.5)
T2	113668 (28.4)
T3	17200 (4.3)
T4	14595 (3.7)
N-stage	
N0	267972 (65.7)
N1	90633 (22.2)
N2	30659 (7.5)
N3	18527 (4.5)
Tumor grade	
I	74612 (16.9)
II	116349 (36.7)
III	141582 (32.1)
Undifferentiated	9203 (2.1)
Unknown	53985 (12.2)
Estrogen receptor expression	
Positive	339967 (77.5)
Negative	99477 (22.5)
Progesterone receptor expression	
Positive	282343(65.6)
Negative	144641(33.6)
Borderline	3332(0.8)
Follow-up time, years	
Mean	8.2
Median (range)	7.6 (0–20.9)
0–5 years	98667(22.3)
5–10 years	213226 (48.3)
>10 years	129838 (29.4)

Table 2 shows a comparison of patient and tumor characteristics based on ER status. Among white patients, 79.7% had an ER-positive tumor, while 62% of black patients had an ER-positive tumor (P<0.0001). Hispanic and Asian patients were less likely than white patients to have an ER-positive tumor but more likely than black patients. Median ages at diagnosis were 63 years for patients with ER-positive cancer and 56 years for patients with ER-negative cancer (*P* = 0.0001). More than 40% of patients who were younger than 40 years at the time of diagnosis had ER-negative disease, while only 29% of patients who were aged between 40–59 years, 25.6% of patients aged 50–59 years, and 17% of patients aged 60 and older (P<0.0001) had ER-negative disease. Patients with a grade III or undifferentiated tumor were more likely to have an ER-negative tumor than patients with grade I or II tumors.

**Table 2:** Comparison of clinicopathologic features by ER status.

Characteristic	ER Negative %	ER Positive %	P value
Race			<0.0001
White	20.3	79.7	
Black	38.0	62.0	
Hispanic	28.1	71.9	
Asian/Pacific Islander	23.5	76.5	
Age at diagnosis, years			0.0001
Mean (Median)	57 (56)	62 (63)	
<40 years	41.1	58.9	<0.0001
40–49	29.0	71.0	
50–59	25.6	74.4	
60 +	17.2	82.8	
T-stage			<0.0001
T1	17.8	82.2	
T2	29.1	70.9	
T3	31.9	68.1	
T4	38.0	62.0	
N-stage			<0.0001
N0	20.8	79.2	
N1	23.8	76.2	
N2	27.4	72.6	
N3	32.6	67.4	
Tumor grade			<0.0001
I	4.6	95.4	
II	11.7	88.3	
III	44.3	55.7	
Undifferentiated	45.2	54.8	
Progesterone receptor expression			<0.0001
Negative	61.0	39.0	
Positive	3.7	96.3	

**Figure 1:**
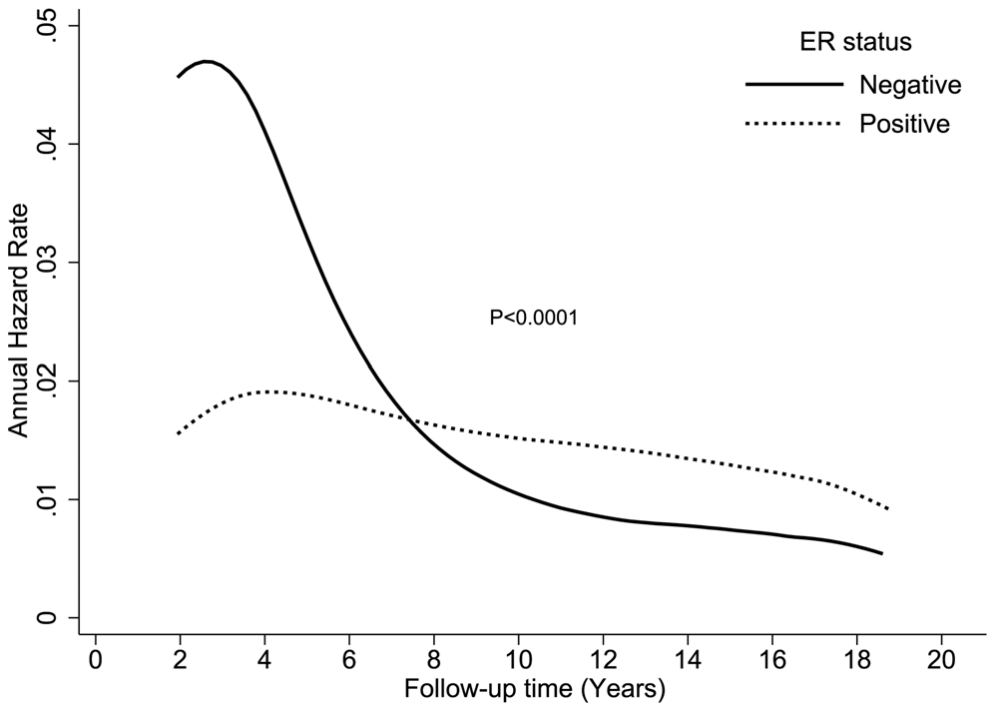
Comparison of disease-specific annual hazard rates by ER status.

Figure 1 shows the DS annual hazard rate by ER status. The DS hazard rate for ER-negative tumors peaked at approximately 5% during year 2, while the DS hazard rate for ER-positive tumors peaked at 2% during year 4. The ER-negative to ER-positive DS hazard rates crossed between years 7 and 8, after which women with an ER-negative tumor had a lower rate of breast cancer death than those with an ER-positive tumor.

**Figure 2:**
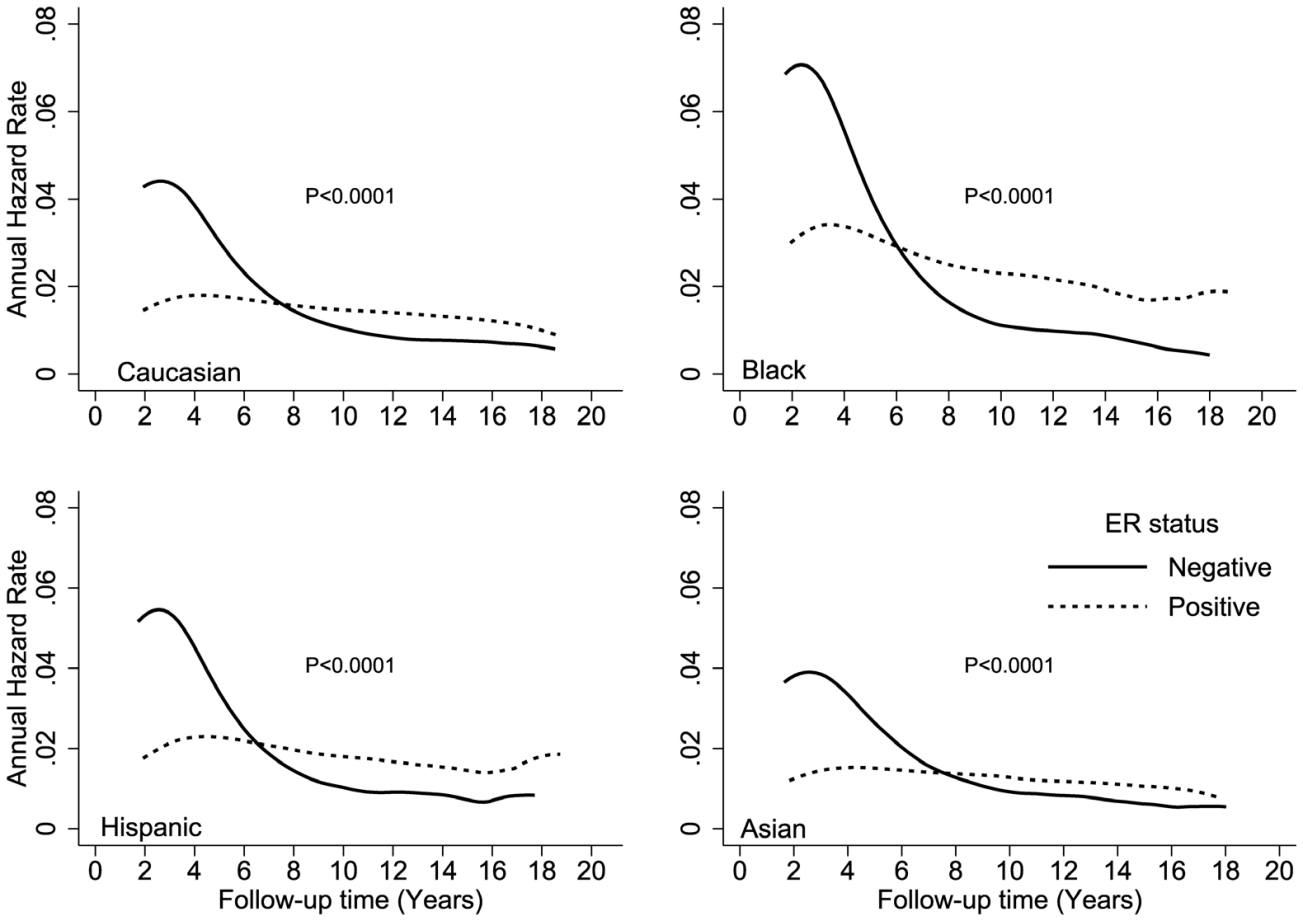
Comparison of disease-specific annual hazard rates by ER status and patient race.

**Figure 3:**
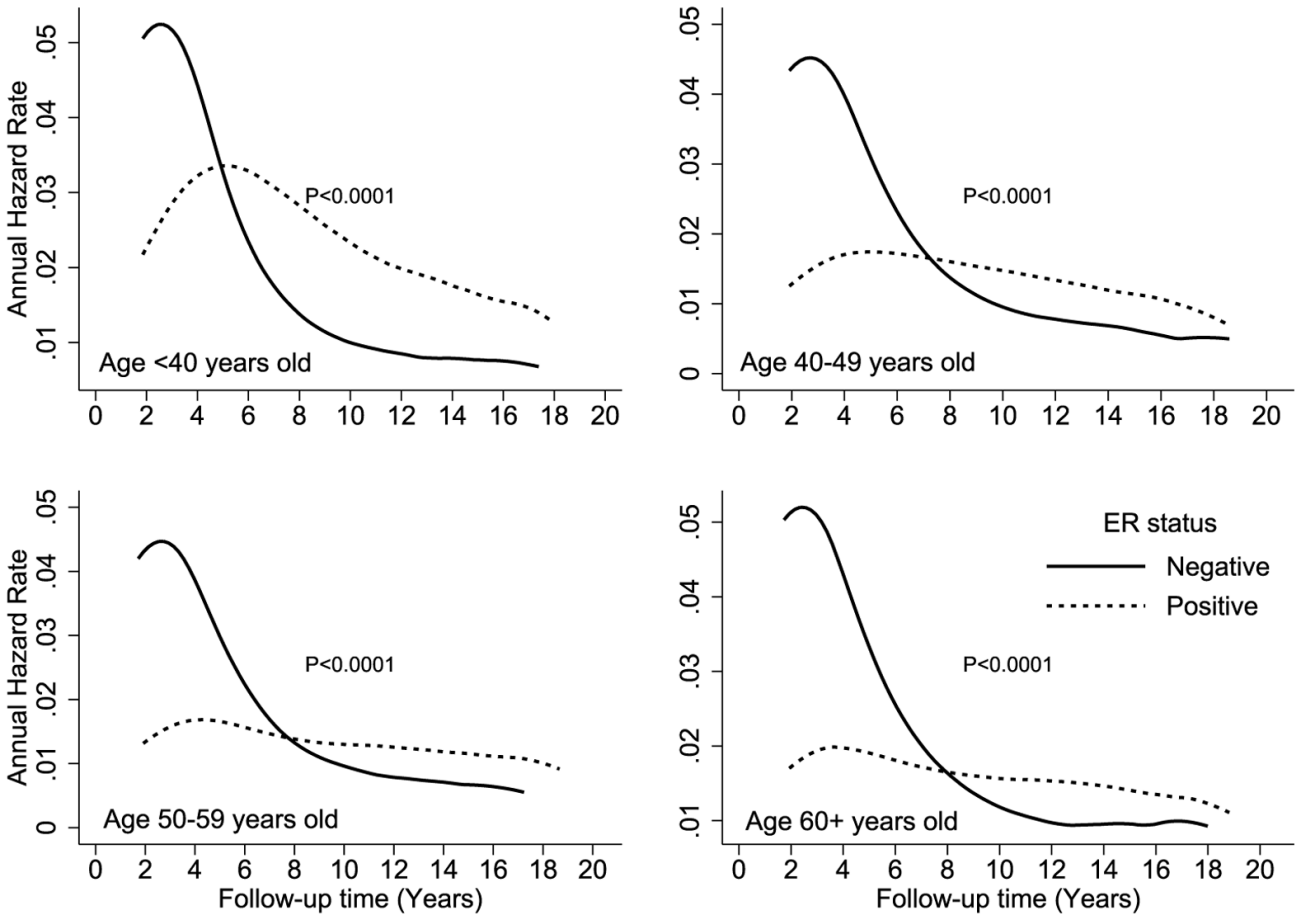
Comparison of disease-specific annual hazard rates by ER status and patient age.

**Figure 4:**
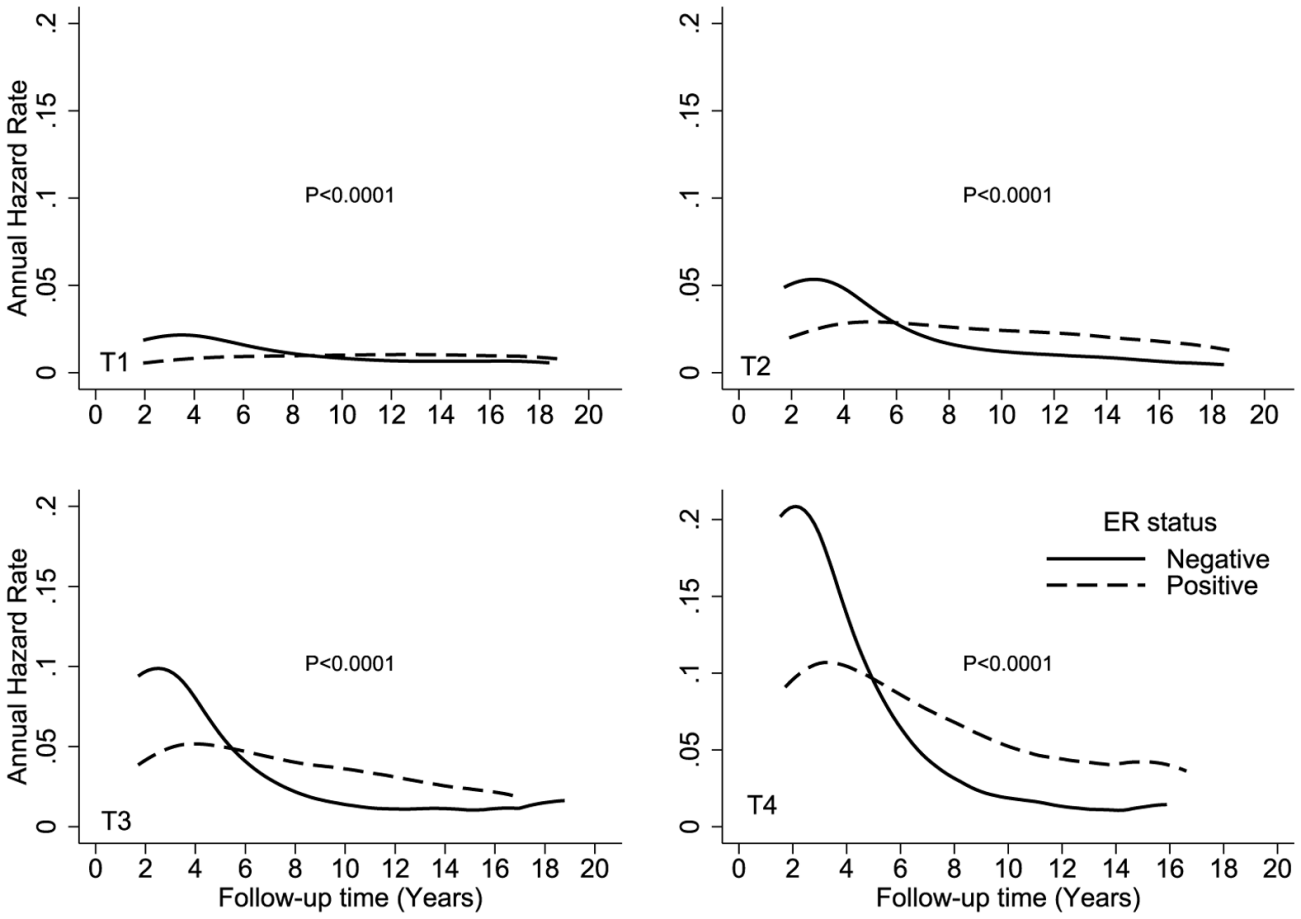
Comparison of disease-specific annual hazard rates by ER status and tumor stage.

**Figure 5:**
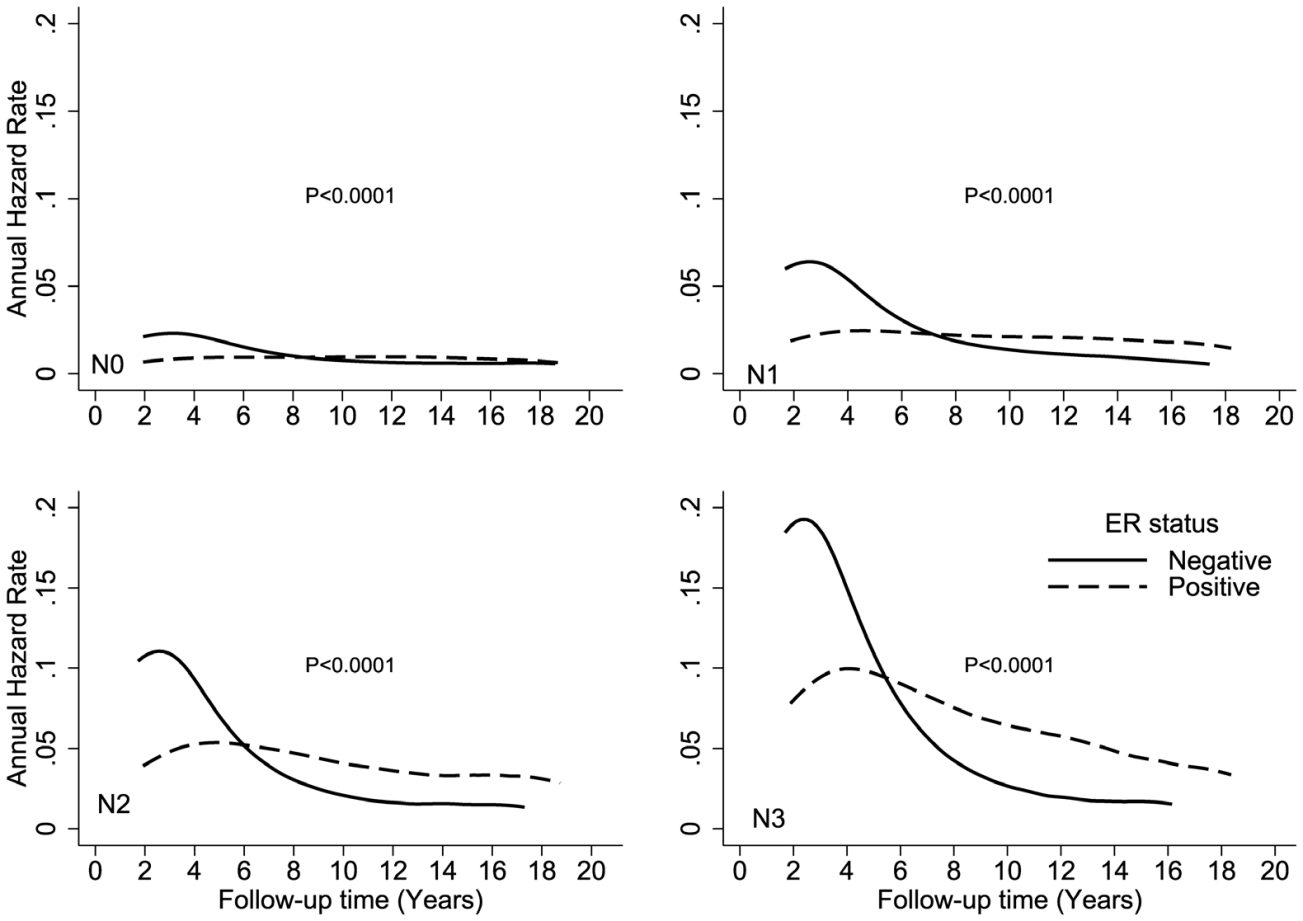
Comparison of disease-specific annual hazard rates by ER status and nodal stage.

**Figure 6:**
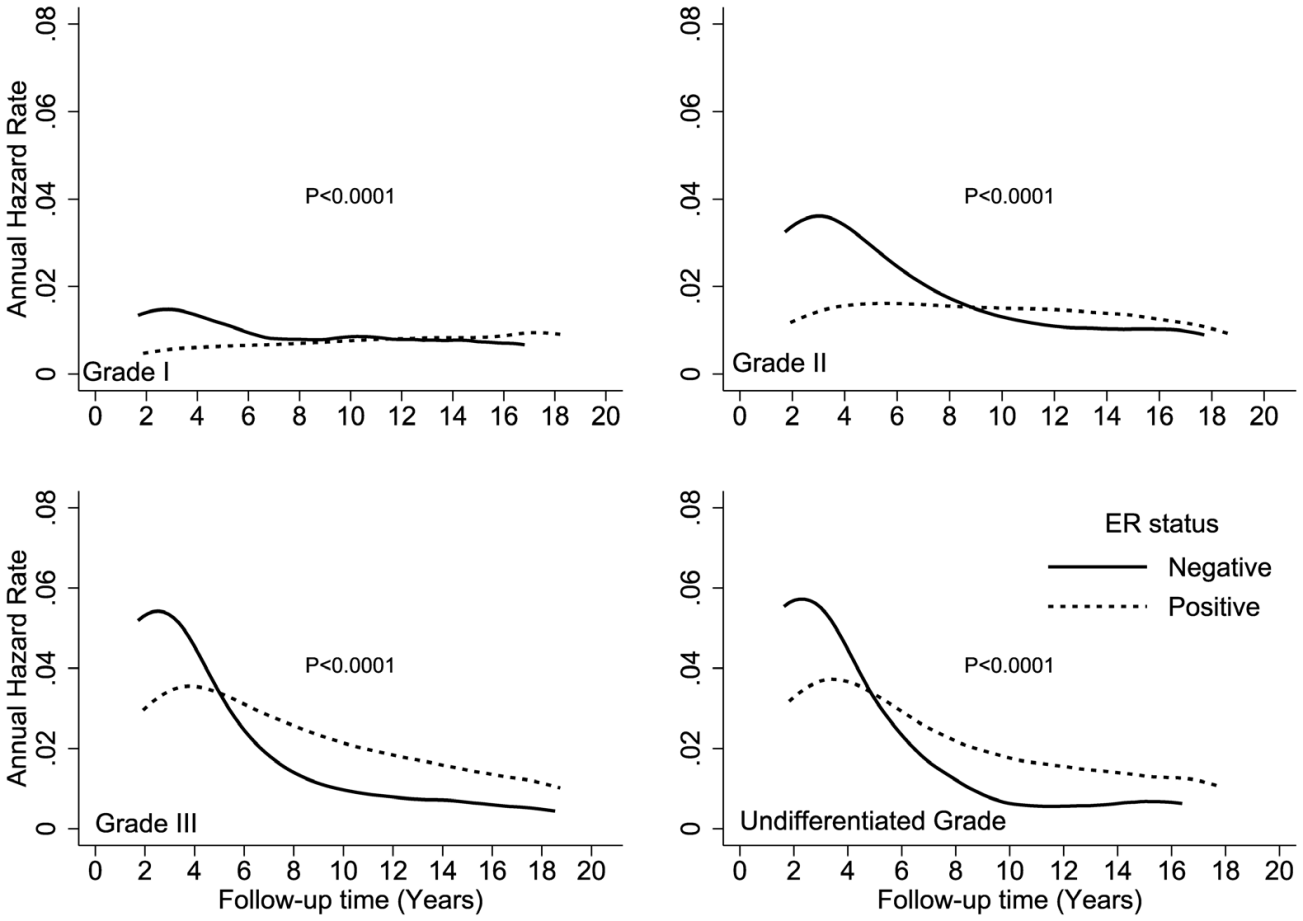
Comparison of disease-specific annual hazard rates by ER status and tumor grade.

Figure 2 shows the hazard rates according to ER expression and race (white, black, Hispanic, and Asian) over time. The crossover in DS hazard rates occurred during year 6 in black and Hispanic patients and during year 8 in white and Asian patients. Black patients had the highest DS hazard rates, with peaks of 7% and 3% at year 2 for ER-negative and ER-positive cancers, respectively. Crossover occurred at year 5 for patients who were younger than 40 years at the time of diagnosis and at approximately year 8 for all other age groups (Figure 3). Younger patients with an ER-positive tumor had an approximately 2-fold higher hazard of DS than older patients. The annual hazard in younger patients increased over the first 4 years and reached a peak between years 4 and 5 after diagnosis. The hazard rate plateaued from years 5 to 6, and then trended downward from year 6 to year 18. Figure 4 shows the annual hazard rate for DS by ER expression and tumor stage. The crossover in DS based on tumor size was greatest for T1 disease, at year 9, and decreased to year 6 in T2/T3 disease and year 5 in T4 disease. A similar pattern is seen with increasing nodal stage (Figure 5). The timing of DS hazard rate crossover also varied by tumor grade. The crossover occurred during year 12 for grade I cancers, year 9 for grade II, year 6 for grade III, and year 5 for undifferentiated tumors (Figure 6).

**Figure 7:**
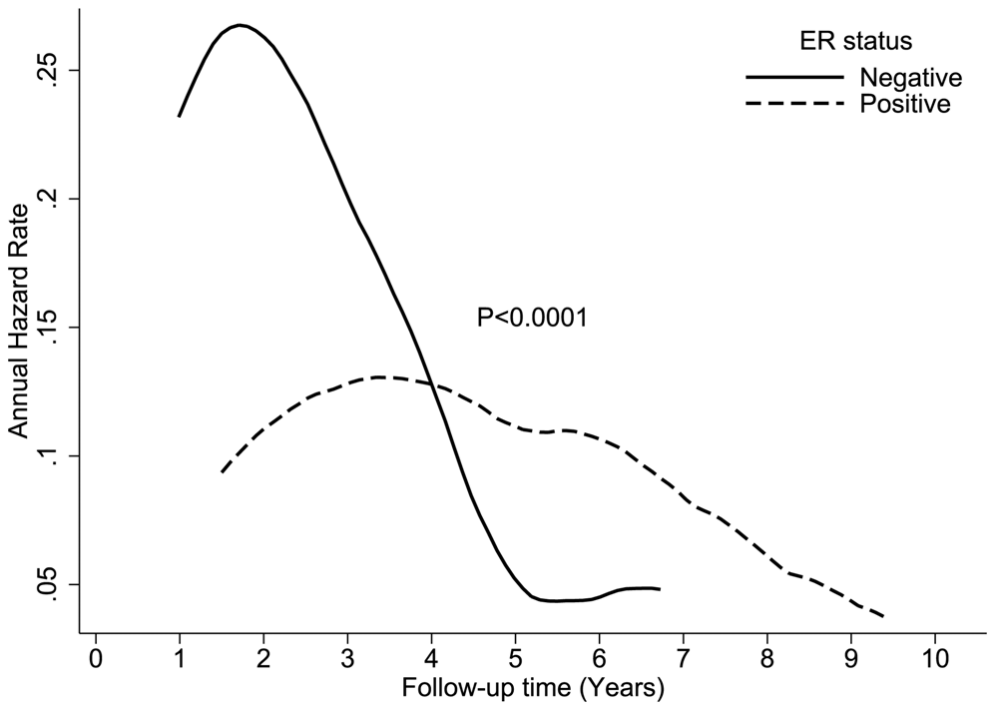
Comparison of disease-specific annual hazard rates by ER status in young (<40 years) black patients who had a T3/T4 tumor with positive nodal disease, grade III or undifferentiated.

When patients were grouped by age, race, tumor and nodal stage, and tumor grade, we found that young (<40 years) black patients who had a T3/T4 tumor with positive nodal disease, grade III or undifferentiated, had the earliest crossover of all groups, at year 4 (Figure 7).

A multivariable Cox proportional hazard model was performed to determine the clinicopathologic factors associated with DS hazard in three different following up periods: 0–5 years, 5–10 years, and>10 years after diagnosis (Table 3). Patients with ER-positive cancer had a better DS hazard (hazard ratio [HR] 0.6, P<0.0001) in the early survival period (0–5 years) than patients with ER-negative cancer. However, patients with ER-positive cancer had a worse DS hazard in the year 5-10 period (HR 1.1, P<0.0001) and the>10 year period (HR 1.37, P<0.00001) than patients with ER-negative cancer. Patients with a higher grade (II/III) or larger (T2/T3) cancer consistently had worse DS hazard at each survival interval than patients with grade I or T1 cancer. Compared to other racial groups, black patients had worse DS hazard in all three survival periods, which increased at each time interval to reach a HR of 1.4 (P<0.0001) at>10 years. Hispanic patients had a slightly worse DS hazard in the>10 year survival interval than in the earlier periods, but this difference was not statistically significant. The risk of dying from breast cancer increased over time for younger patients, whereas this pattern was reversed in older patients.

**Table 3:** Multivariable Cox proportional hazards analysis of clinicopathologic factors associated with death by breast cancer.

	Following time 0–5 year (N* = 350998)				Following time 5–10 year (N* = 283336)				Following time>10 year (N* = 99976)			
Factor	HR	P	95% CI		HR	P	95% CI		HR	P	95% CI	
Estrogen receptor												
Negative	Referent				Referent				Referent			
Positive	0.66	<0.0001	0.65	0.67	1.1	<0.0001	1.09	1.14	1.37	<0.0001	1.3	1.4
Tumor grade												
I	Referent				Referent				Referent			
II	1.8	<0.0001	1.7	1.9	1.9	<0.0001	1.8	2.0	1.6	<0.0001	1.4	1.7
III	3.0	<0.0001	2.8	3.1	2.4	<0.0001	2.2	2.5	1.4	<0.0001	1.3	1.6
Undifferentiated	3.1	<0.0001	2.9	3.4	2.2	<0.0001	2.0	2.5	1.3	0.01	1.2	1.5
Race												
White	Referent				Referent				Referent			
Black	1.5	<0.0001	1.4	1.5	1.3	<0.0001	1.2	1.4	1.4	<0.0001	1.2	1.6
Hispanic	1.0	0.2	0.98	1.1	1.0	0.6	1.0	1.1	1.1	0.1	1.0	1.2
Asian	0.84	<0.0001	0.8	0.87	0.9	<0.0001	0.8	0.9	0.9	0.2	0.8	1.0
T-stage												
T1	Referent				Referent				Referent			
T2	2.1	<0.0001	2.0	2.2	1.7	<0.0001	1.6	1.8	1.5	<0.0001	1.4	1.6
T3	3.2	<0.0001	3.1	3.4	2.0	<0.0001	1.9	2.2	1.5	<0.0001	1.3	1.7
T4	5.4	<0.0001	5.2	5.6	2.9	<0.0001	2.7	3.1	1.7	<0.0001	1.4	2.1
N-stage												
N0	Referent				Referent				Referent			
N1	1.9	<0.0001	1.8	2.0	1.8	<0.0001	1.7	1.9	1.9	<0.0001	1.8	2.0
N2	3.0	<0.0001	2.9	3.1	3.2	<0.0001	3.1	3.4	2.9	<0.0001	2.6	3.2
N3	4.7	<0.0001	4.5	4.8	4.6	<0.0001	4.4	4.9	4.0	<0.0001	3.5	4.5
Age at diagnosis, years												
<40 years	Referent				Referent				Referent			
40–49	0.9	<0.0001	0.8	0.9	0.8	<0.0001	0.7	0.8	0.8	<0.0001	0.7	0.9
50–59	0.9	<0.0001	0.9	0.95	0.7	<0.0001	0.7	0.8	0.9	0.02	0.8	0.98
60 +	1.4	<0.0001	1.3	1.4	1.0	0.3	1.0	1.1	1.1	0.02	1.02	1.3

Asian: Asian/Pacific Islander; * N is the number of individuals with complete data for all the factors included in the Cox-model which are at risk at the beginning of each interval.

**Figure 8:**
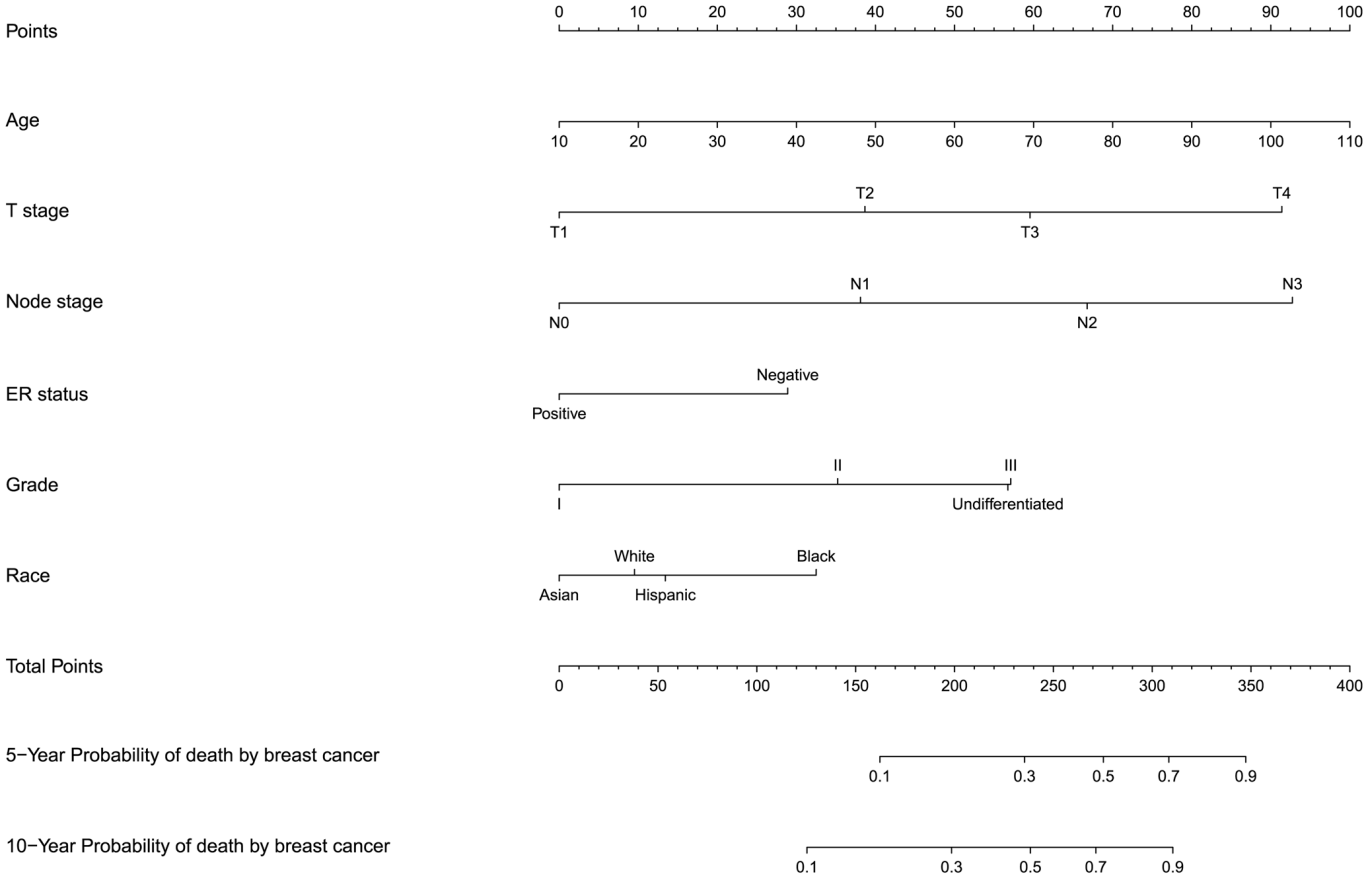
Nomogram for predicting 5- and 10-year probability of death by breast cancer after surgery. To estimate risk, calculate points for each variable by drawing a straight line from patient's variable value to the axis labeled “Points.” Sum all points and draw a straight line from the total point axis to the 5- and 10-year probability of death by breast cancer axis.

A multivariable Cox proportional hazard model was built to determine the clinicopathologic factors associated with death from breast cancer in whole cohort (table 4). Older patients, patients with ER negative tumor, advanced tumor stage, positive lymph nodes, or with higher tumor grades had a higher probability of death from breast cancer. A nomogram based on the Cox regression model is shown in figure 8. The nomogram predicts the probability that the patient will die from breast cancer within 5 or 10 years from the date of surgery. As an example, the nomogram predicts that a 50-year-old patient (40 points) with stage T1 (0 points), N0 (0 point), ER positive (0 points), grade I (0 points) tumor has a lower than 10% risk (total points, 40) of death from breast cancer within 10 years of surgery. Conversely, 50-year-old patient (40 points) with stage T4 (90 points), N3 (90 point), ER negative (28 points), grade III (55 points) tumor has a more than 70% risk (total points, 300) of death from breast cancer within 10 years of surgery. Tumor and nodal stage were higher ranks of predictors than others since the points were higher than other factors.

**Table 4:** Multivariable Cox proportional hazards analysis of clinicopathologic factors associated with death by breast cancer.

Factor	HR	P	95% CI	
Estrogen receptor				
Positive	Referent			
Negative	1.6	<0.0001	1.58	1.65
Tumor grade				
I	Referent			
II	1.8	<0.0001	1.7	1.9
III	2.6	<0.0001	2.5	2.7
Undifferentiated	2.6	<0.0001	2.4	2.7
Race				
White	Referent			
Black	1.5	<0.0001	1.4	1.5
Hispanic	1.1	<0.0001	1.03	1.1
Asian	0.9	<0.0001	0.8	0.9
T-stage				
T1	Referent			
T2	1.9	<0.0001	1.86	1.94
T3	2.7	<0.0001	2.6	2.8
T4	4.6	<0.0001	4.4	4.7
N-stage				
N0	Referent			
N1	1.9	<0.0001	1.8	2.0
N2	3.0	<0.0001	2.9	3.1
N3	4.7	<0.0001	4.5	4.8
Age at diagnosis, years	1.02	<0.0001	1.01	1.02

C-index: 0.781, CPE: 0.71, Asian: Asian/Pacific Islander; Age was treated as continues variable.

## Discussion

Our study used the large, population-based SEER data to show differences in DS hazard rates for patients with ER-positive breast cancer or ER-negative cancer as related to patient age and race, tumor and nodal stage, and tumor grade. ER-positive cancers were more common in white patients and in older patients. Patients with an ER-positive tumor were more likely to have a lower grade tumor and local disease than patients with an ER-negative tumor. Our findings are consistent with previous reports [7]–[9] in that patients with an ER-negative breast cancer are more likely to die of their disease at an earlier time than those with an ER-positive cancer. In all subsets, with increasing time from diagnosis, crossover occurs such that patients with ER-negative cancer have a better DS hazard rate than those with ER-positive cancer.

Our results show that, after 7 years, ER-positive cancers cross over to have a higher DS annual hazard rate than ER-negative cancers, and this difference persists through 18 years. Furthermore, we identified specific patient groups with earlier ER-positive and -negative crossover. Patients younger than 40 years with ER-positive cancer had an earlier crossover (at year 4) than other age groups. Time to crossover also varied among different racial groups. Our finding that black and Hispanic patients had earlier crossover (year 6) than white and Asian patients (both, year 8) is consistent with previously published studies [6], [9].

The National Surgical Adjuvant Breast and Bowel Project (NSABP) B-14 trial showed no significant benefit for tamoxifen beyond 5 years of treatment in node-negative breast cancer [14]. Seven-year disease-free survival rate was actually slightly higher in patients who took the standard 5 years of tamoxifen followed by placebo than in patients taking tamoxifen for an additional 7 years (82% vs. 78%, P = 0.03). Overall, there were no differences in relapse-free survival or overall survival in this study. When evaluated by patient age, there was a slight benefit in overall survival but not in recurrence-free survival or disease-free survival in patients 49 years and younger who took additional tamoxifen than in those who took tamoxifen for 5 years. There was no demonstrable benefit from additional tamoxifen in recurrence-free, disease-free, or overall survival among women older than 50 years.

The recently reported ATLAS and aTTom (Adjuvant Tamoxifen – To Offer more) trials found a further reduction in breast cancer recurrence and increase in survival rate 10 years after diagnosis in patients who received 10 years of tamoxifen compared to patients who received 5 years of this treatment [11], [15]. The ATLAS trial demonstrated a benefit regardless of patient age or menopausal status, in node-negative as well as node-positive disease. However, there were more side effects with 10 years of tamoxifen, including uterine cancer (HR 1.74) and pulmonary embolus (HR 1.87). The trial was also limited by the small number of premenopausal patients (8–10%) and the large number of patients in whom the ER status was unknown (37%). The aTTom trial showed similar results, with reduced breast cancer mortality with longer tamoxifen treatment (10 years) in patients with ER-positive or ER-untested cancers.

The American Society of Clinical Oncology clinical practice guideline published in 2010 recommends that women who receive extended adjuvant endocrine therapy should have a total of 8-10 years of treatment, 5 years of tamoxifen followed by 3-5 years of an aromatase inhibitor [16]. The current National Comprehensive Cancer Network guidelines (NCCN V 3.2013) recommends 10 years of tamoxifen for invasive hormone receptor–positive breast cancer in premenopausal woman or 5 years of tamoxifen and 5 years of an aromatase inhibitor if she experiences menopause during treatment (http://www.nccn.org/professionals/physician_gls/pdf/breast.pdf, accessed 4/9/2014).

Given the recommendations for extended duration of endocrine therapy, questions regarding eligibility for more treatment as well as compliance and persistence with treatment have become a focus of interest [17]–[20]. Myrick *et al*. reported that only 48.3% of the patients who began endocrine therapy were eligible for extended therapy, and patients who were offered/recommended to receive extended therapy had a compliance rate of 84.7% and a persistence rate of 72% [20]. The Patient's Anastrozole Compliance to Therapy program reported an 88% compliance rate and 40% persistence rate when educational materials were distributed with adjuvant anastrozole [18]. Neven *et al*. also reported higher compliance rates in patients receiving educational materials [21].

Clinicians should consider and compare the benefits of endocrine therapy with the cost and side effects. Tamoxifen is the least expensive of all the endocrine therapies; a generic version costs about $100/month in the USA, according to Susan G. Komen for the Cure (http://ww5.komen.org/uploadedfiles/content_binaries/806-326a.pdf Access 3/1/2014). Aromatase inhibitors usually cost significantly more than tamoxifen [22]. The side effects of tamoxifen include vasomotor symptoms, gynecologic symptoms, sexual dysfunction, and increased rates of endometrial cancer, stroke, pulmonary embolism, and deep vein thrombosis [23], [24]. Aromatase inhibitors are better tolerated, with fewer side effects, but are associated with increased risk of osteopenia, osteoporosis, and fractures [25].

Because the present study is a retrospective population-based study, data regarding socioeconomic status, family history of breast cancer, lifestyle factors, HER2 status, and administration of and compliance with neoadjuvant or adjuvant systemic therapies were limited. These factors therefore could not be evaluated as potential confounders or effect modifiers of the relationships observed. Despite these limitations, the strengths of this study include the large sample size and the most comprehensive analyses yet undertaken of ER-positive and -negative crossover on hazard of DSS by patient race and age, tumor and nodal stage, and tumor grade.

In conclusion, we found that the timing of crossover and DS hazard rates varied depending on the patient's clinical and disease factors. Patients who were black or Hispanic, younger than 40 years, or had a larger tumor, nodal disease, or a higher grade tumor had an earlier crossover of death hazard for ER-positive or ER-negative disease. Young (<40 years) black patients who had a T3/T4 tumor with positive nodal disease, grade III or undifferentiated, had the earliest crossover of all groups, at year 4. These findings may help guide recommendations regarding the duration of endocrine therapy for patients with ER-positive cancer.
